# Do CAR-T and Allogeneic Stem Cell Transplant Both Have a Place in Lymphoid Neoplasms?

**DOI:** 10.3390/ijms24021045

**Published:** 2023-01-05

**Authors:** Massimo Martino, Filippo Antonio Canale, Virginia Naso, Gaetana Porto, Demetrio Gerace, Alessandro Allegra

**Affiliations:** 1 Stem Cell Transplant and Cellular Therapies Unit, Great Metropolitan Hospital “Bianchi-Melacrino-Morelli”, 89133 Reggio Calabria, Italy; 2 Stem Cell Transplant Program CIC 587, Great Metropolitan Hospital “Bianchi-Melacrino-Morelli”, 89133 Reggio Calabria, Italy; 3 Division of Hematology, Department of Human Pathology in Adulthood and Childhood “Gaetano Barresi”, University of Messina, 98125 Messina, Italy

**Keywords:** allogeneic stem cell transplantation, autologous stem cell transplantation, CAR-T treatment, lymphoproliferative diseases, acute lymphoblastic leukemia, mantle cell lymphoma, diffuse large B cell lymphoma, relapsed lymphoma

## Abstract

Allogeneic stem cell transplantation (allo-SCT) represented the first immunotherapy to treat hematologic malignancies: it has been considered as a cure for the disease and never as an approach to extend the life of patients. The success of allo-SCT derives both from the ability to treat patients with intensive chemoradiotherapy and from the potent graft-versus-leukemia effects mediated by donor immunity. Although considerable progress has been made in the last years, significant barriers still remain in the form of disease relapse, graft-versus-host disease, infectious complications, and regimen-related toxicities. Moreover, the treatment of hematologic malignancies, particularly acute lymphoblastic leukemia and certain forms of lymphomas, has been revolutionized by the commercial introduction of genetically modified autologous T-lymphocyte therapy (CAR-T). Our review discusses current standards and the shifting paradigms in the indications for allo-SCT and the role of CAR-T cell therapy for lymphoid neoplasms.

## 1. Introduction

### General Considerations on Allogenic Stem Cell Transplants and CAR-T Cells

Allogeneic stem cell transplantation (allo-SCT) has developed the concept of immunotherapy as a tool against cancer and has made ground-breaking progress in treating many malignant diseases [1]. The success of transplantation derives from intensive chemoradiotherapy and the ability to treat patients with an immunological effect known as “graft-versus-leukemia” [2]. Tailored conditioning regimens, improved donor selection, better selection of patients, and supportive care have contributed to the reduction of transplant-related mortality and morbidity [3]. The possibility of searching for a donor in registries and developing haploidentical platforms ensures that the transplant procedure can be offered to all who need it [4,5]. Despite technological progress that has significantly improved patient survival, allo-SCT carries a significant risk of immunological complications, such as Graft-Versus-Host Disease (GVHD), regimen-related toxicities, infectious complications, and disease relapse.

The year 2018 represents a historic moment for cancer immunotherapy: the first two products containing chimeric antigen receptor (CAR)-T cells, tisagenlecleucel and axicabtageneciloleucel, were approved for commercial use, revolutionizing the treatment landscape for relapsed or refractory (R/R) B acute lymphoblastic leukemia (ALL) and R/R B-cell non-Hodgkin lymphoma (NHL) [6,7,8]. Subsequently, regulators approved lisocabtagene maraleucel in R/R B-cell NHL [9], brexucabtagene autoleucel in R/R mantle cell lymphoma (MCL) [10] and adult patients with R/R B-ALL [11], idecabtagene vicleucel [12] and ciltacabtagene autoleucel in R/R multiple myeloma (MM) [13]. Table 1 summarizes the main characteristics of the constructs and the clinical indications.

**Figure 1:**
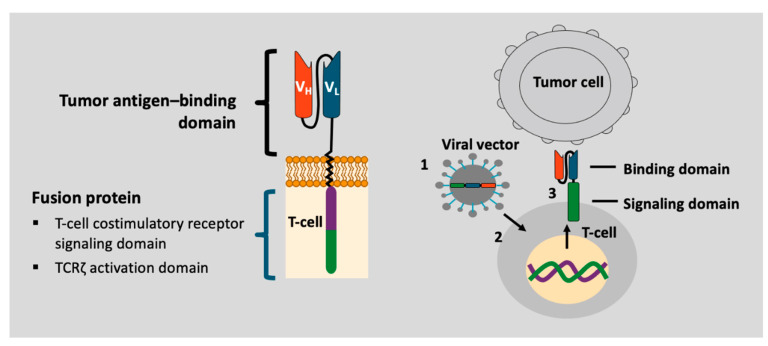
CD19 and BCMA-directed CAR T-cells comprising a CD19 or a BCMA antigen-binding domain, a costimulatory domain (generally CD28 or 4-1BB), and a CD3-ζsignaling domain.

CARs are synthetic proteins expressed on the surface of T cells. They consist of intracellular and extracellular components [14]. The intracellular domains ensure intracellular signaling to activate the effector functions of the CAR-T cells; the extracellular part is an antigen-recognizing domain composed of fragments of monoclonal antibodies that recognize a specific protein on the surface of malignant cells (e.g., CD19 on B-cells). First-generation CAR-T cells utilized an intracellular domain from the CD3 ζ-chain of the T-cell receptor (TCR), which induced cytotoxicity against targeted malignant cells but failed to support CAR-T cells expansion in vivo after reinfusion. Second- and third-generation CAR-T cells have an additional costimulatory intracellular domain (e.g., CD28, 41BB, OX40) that enhances the CAR-T cells’ ability to proliferate, expand, and persist in vivo (Figure 1). However, new strategies are needed to increase the safety profile and shorten the manufacturing process during CAR-T cells therapy production [15].

Both allogeneic transplantation and CAR-T are being developed as therapies that could cure neoplastic disease. The question that clinicians are trying to answer is whether the two treatments are complementary to each other or are therapeutic alternatives. In this article, we report the clinical interplay between allo-SCT and CAR-T therapy in lymphoid neoplasms and the lessons learned from each. In addition, we have considered lymphoid diseases where CAR-Ts are available for commercial use in Europe, excluding MM because allo-SCT is in disuse in this disease [5].

## 2. allo-SCT and CAR-T in Lymphoid Neoplasms

### 2.1. R/R DLBCL

allo-SCT has been considered a curative treatment option for patients with DLBCL who relapse or progress after auto-SCT [16]. A registry study reported results on 101 patients who received an allo-SCT for DLBCL between 1997 and 2006 [17]. Three-year non-relapse mortality (NRM), relapse rate (RR), progression free survival (PFS), and overall survival (OS) were 28.2%, 30.1%, 41.7%, and 53.8%, respectively. No statistically significant differences were seen between patients transplanted after a myeloablative (MAC) or a reduced-intensity conditioning (RIC), or patients transplanted from mismatched related or matched unrelated donors. A prospective randomized clinical trial compared GVHD prophylaxis including rituximab, after allo-SCT [18]. At a median follow-up of 4.0 years, no significant difference was found between patients receiving or not receiving rituximab in addition to standard GVHD prophylaxis. In all patients, the 1-year OS rate was 52%, with significant differences between patients transplanted from matched family donors or unrelated donors not receiving or receiving anti-thymocyte globulin for GVHD prophylaxis, where the OS was 64.7%.

A study evaluated 121 patients with R/R B cell lymphomas who underwent an allo-SCT after an RIC regimen [19]. All the patients had failed a previous auto-SCT. At a median follow-up of 41 months, the 3-year PFS and OS were 50% and 61%, respectively. Long-term outcome was also evaluated with the composite endpoint of GVHD-free and relapse-free survival (GRFS). The 1-year and 3-year GVHD-free and GRFS were 40% and 34%, respectively. The conclusions were that allo-SCT could cure a fraction of these patients.

The Center for International Blood and Marrow Transplant Research (CIBMTR) performed a registry analysis of DLBCL patients undergoing allo-SCT after a failed prior auto-SCT [20]. The results were as follows: NRM was 23% and 30% at 1 and 3 years respectively; MAC provided no benefit; and GVHD increased the risk of non-relapse and overall mortality without reducing the risk of relapse/progression. The authors indicated a prognostic model to predict PFS after allo-SCT, with 5 points for chemoresistance, 4 points for a Karnofsky performance score <80, and 2 points for patients who went from auto-SCT to allo-SCT in less than one year. The 3-year OS was 43% in patients with 0 points (low risk), 39% in those with 2–5 points (intermediate risk), and 19% and 11% in those with 6–9 points (high risk), and 11 points (very high risk), respectively.

These data show that allo-SCT is a valid alternative to any other treatment for patients relapsing after failure from auto-SCT, and allo-SCT should be considered for patients with early relapse after first-line chemotherapy [16].

Auto-SCT remains the standard of care in a late chemosensitive relapse of DLBCL [5,16]. CAR-T is currently considered the standard of care in the third line, according to clinical trial and real-world date results where axicabtagene ciloleucel, tisagenlecleucel, and lisocabtagene maraleucel have produced significant rates of overall response rate (ORR) and complete remission (CR) [21,22,23,24] (Table 2).

**Figure 2:**
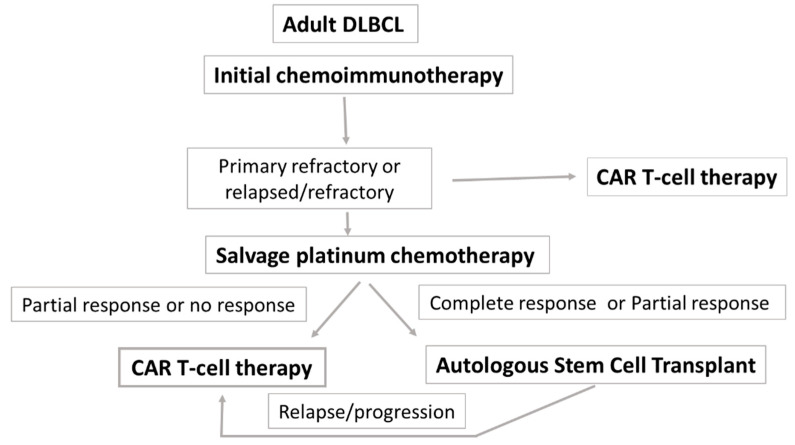
Fitting CAR-T Cell Therapy Into Current Treatment Paradigms in DLBCL.

In patients who are refractory or at high risk of relapse, the debate is wide open as to whether the treatment of choice should be CAR-T. High-risk relapse of DLBCL has been defined as a remission duration of <12 months after first-line therapy [16]. The new European Society for Blood and Marrow Transplantation (EBMT) guidelines indicate CAR-T as the therapy of choice in this category of patients [16] based on the results of two recent phase III clinical studies [25,26]. Due to the unique characteristics of the study design, the chemosensitivity of the patients in the CAR-T arms was unknown. The EBMT guidelines introduced a category defined as untested relapse; for this situation, the results of CAR-T therapy have been considered superior to those of standard chemotherapy followed by auto-SCT. For patients with high-risk R/R LBCL and unknown chemosensitivity, anti-CD19 CAR-T should replace auto-SCT as the standard care. Figure 2 summarizes the inclusion of CAR-T cell therapy in the current treatment paradigms for DLBCL.

The role of allo-SCT appears to be significantly reduced, at least in patients with refractory, high-risk, or relapsed disease after auto-SCT [27]. Moreover, comparing CAR-T cell therapy to allo-SCT using the CIBMTR prognostic tool, the probability of a better PFS was higher for CAR T-cell therapy [28]. A multivariate evaluation of chemoresistance before transplant and its impact on outcomes showed that patients with chemo-resistant disease had a higher risk of NRM, progression, and relapse. Patients with a Karnofsky performance status <80 and chemo-resistant condition had inferior OS outcomes.

Limited data exist to document the safety and efficacy of allo-SCT after CAR-T therapy, and most of the evidence has come from a small patient series. Zurko et al. [29] presented data for patients treated at 18 US academic medical centers. The 88 patients included in the analysis had a median age of 54. About half the patients were in CR before allo-SCT, and a fourth had partial response (PR). The median duration from CAR-T failure to allo-SCT was 8.4 months. The 1-year NRM, PFS, and OS were 22%, 45%, and 59%, respectively. By multivariate analysis, patients in CR at allo-SCT had better OS than patients in PR. The investigators found no predictors of PFS, NRM, or progression/relapse. Additionally, patients who had received fewer than two lines of therapy between CAR-T failure and allo-SCT fared better than those who received two or more intervening lines of treatment.

Another study evaluated the role of allo-SCT after CAR T-cell therapy in 39 adults LBCL patients [30]. The disease status at allo-SCT was CR (41%), PR (38%), or progressive disease (PD) (21%). In addition, the 2-year NRM and relapse/progression incidences were 26% and 43%, respectively. With a median follow-up of 32 months, the 2-year OS and PFS were 45% and 31%, respectively.

Based on these findings, in patients with DLBCL treated with CAR-T cells and in whom this therapy has failed, allo-SCT may be an option, at least for those who achieve a CR after CAR-T failure. The data may help inform decisions related to one of the critical challenges of CAR T-cell therapy. Although the treatment produces response rates as high as 83% in R/R LBCL [21,22,23,24], responses are durable in only 30–40% of cases and the median OS after CAR-T failure is poor [31,32].

### 2.2. MCL

**Figure 3:**
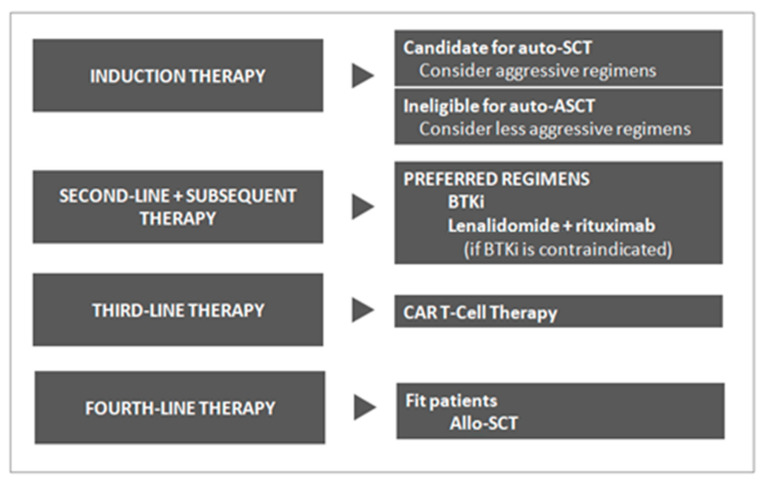
Fitting CAR T-Cell Therapy Into Current Treatment Paradigms in MCL.

The survival of patients with MCL has improved since the introduction of rituximab and other novel agents, such as BTK inhibitors, in the overall treatment landscape [33,34]. For younger MCL patients, various intensive chemotherapy regimens incorporating rituximab and high-dose cytarabine are optimal, and auto-SCT remains a highly efficacious initial therapy [35]. Unfortunately, most patients with MCL relapse even after achieving a CR to first-line therapy. In the relapsed setting, the first-line treatment of choice is usually a BTK inhibitor [34]. BTK inhibitors work very well in relapsed MCL with high response rates and good tolerability profiles. However, the length of remission for patients receiving BTK inhibitors tends to be approximately 1.5–2.0 years [36]. The available evidence does not suggest a benefit of allo-SCT in CR1, and upfront allo-SCT, outside of clinical trials, is not recommended [37] (Figure 3).

CAR T-cell therapy could be a major breakthrough in MCL. The EMA recently approved brexucabtagene autoleucel for third-line treatment, which is a CAR T-cell therapy directed against CD19, for R/R MCL based on data from the ZUMA-2 trial [38]. The ORR was 92%, and 67% at a median 17.5-month follow-up. However, we need longer follow-up to know what the longer-term efficacy is with this agent. As with all CAR T-cell therapy, brexucabtagene autoleucel is associated with the risk for cytokine release syndrome (CRS) and immune effector cell-associated neurotoxicity syndrome (ICANS), so treatment with this agent is not without some risk. However, relapsed MCL has historically been a difficult disease to manage, and therefore, we can accept a certain amount of toxicity for an agent with good efficacy.

### 2.3. The Rationale for Clinical Development of CAR T-Cell Therapy in ALL

Most adults with ALL who achieve CR will relapse, and the prognosis of these patients is poor [39]. However, treatment options for patients with R/R ALL are rapidly expanding with the advent of promising immunotherapy. Inotuzomab ozogamicin is a humanized monoclonal antibody targeting CD22 that is conjugated to calicheamicin, a cytotoxic compound that binds to the minor groove of DNA and causes double-stranded DNA breaks. Inotuzumab has been studied for the treatment of ALL in R/R disease in the upfront setting [40]. The drug is well tolerated with easy administration and a CR rate of 80%. A potential disadvantage is the increased risk of sinusoidal obstruction syndrome (VOD) in prior allo-SCT and liver disease, and that most data are from the s1/2 setting only.

Blinatumomab is a bispecific T cell-engaging antibody drug that redirects cytotoxic T cells to cells expressing CD19 and is approved for R/R ALL [41]. The antibody itself contains the variable domains of CD19 and CD3, which are linked together. Once bound to CD19 as part of the antibody complex, cytotoxic T cells induce cell death via the perforin system. Blinatumomab is manageable, with reversible AEs, and a CR rate between 40% and 50%; moreover, it is highly effective in a minimal residual disease (MRD) + setting, with a CR rate of 80%. However, the drug is less effective with high bone marrow blasts, and the need for a continuous infusion/pump is a disadvantage. Despite blinatumomab and inotuzumab treatment, the median OS for R/R B-ALL remains low at 7–8 months [42,43].

Anti-CD19 CAR-T cell therapy is approved for patients up to 25 years of age based on the ELIANA study [44] and, more recently, for adult ALL based on the ZUMA-3 trial [45].

ELIANA was an international, open-label, single-arm phase II study that enrolled 92 patients aged 3–21 years with R/R B-cell ALL. After tisagenlecleucel infusion, ORR at 3 months was 81%, and EFS and OS at 6 and 12 months were 73%, 50%, 90%, and 76%, respectively.

Zuma-3 was a multicenter, open-label phase I/II trial that enrolled adult patients with R/R B-cell ALL. After a lymphodepletion with fludarabine + cyclophosphamide, 55 patients received Brexucabtagene autoleucel at a dose of 1 × 10^6^ cells/kg. The ORR was 70.9% and the CR rate was 56.4%. At the median follow-up of 16.4 months, 39 patients had CR or CR with incomplete hematological recovery. The median duration of remission, RFS and OS were 12.8, 11.6 and 18.2 months, respectively. The median OS was not reached among responders, and 38 (97%) patients had MRD negativity.

Most trials of CAR-T cells in R/R ALL demonstrate impressive response rates, with >70% of patients achieving CR regardless of cytogenetic background, prior therapies, or age [46]. Prognostic factors associated with higher remission rates and better outcomes in adult ALL include lower disease burden (assessed by bone marrow blast count), lower LDH, and higher platelet count before lymphodepletion, whereas TP53 mutations are associated with worse outcomes [47,48]. CD19 CAR T-cells can induce high CR rates, even in patients with blinatumomab/inotuzumab failure and with multiple prior treatments [46,47,48]. The potential disadvantages are the need for bridging time during cell manufacturing, CRS and ICANS. The early trials showed that a subset of patients could be cured with no additional therapy, but relapse after CD19 CAR T-cell therapy remains challenging and a clinically unmet need.

Summers et al. showed that a consolidation with allo-SCT in pediatric and young adult subjects following CAR-T cell-induced remission improved LFS in patients with no previous history of allo-SCT and those with short functional CAR-T cell persistence [49].

In a pediatric single-center study, patients in MRD-negative CR after CAR-T and who underwent a subsequent allo-SCT showed better EFS and OS [50]. Another single-center study in adults found no difference in outcomes for patients bridged to allo-SCT versus observation [51]. Most pediatric patients treated with tisagenlecleucel were not bridged to allo-SCT, and a subset had durable remissions that correlate with in vivo functional persistence. However, the outcomes of initial bridge to allo-SCT versus observation are unknown [44].

Park et al. published the most extensive clinical series about the role of allo-SCT after CAR-T in R/R B-ALL patients [52]. The authors identified 347 patients with a median age of 13 years who received CD19 CAR T cells. With a median follow-up of 12.7 months, DFS at 3, 6 and 12 months following CAR T cell infusion was 80.9%, 71.2%, and 57.6%, respectively. The OS at 3, 6 and 12 months was 93.6%, 89.8%, and 79.4%, respectively. Incidences of relapse without censoring at subsequent allo-SCT at months 3, 6, and 12 were 18.5%, 28.2%, and 40.6%, respectively. Of the 347 patients who received CD19 CAR therapy, 62 (18%) had subsequent allo-SCT in CR as a consolidation post-CAR with a median from CAR T to allo-SCT of 4.7 months. Among the 57 patients with MRD evaluation, 56 were negative (98%). allo-SCT resulted in a significant reduction in leukemia relapse with a trend towards better DFS with a favorable safety profile of 8.9% TRM rate.

**Figure 4:**
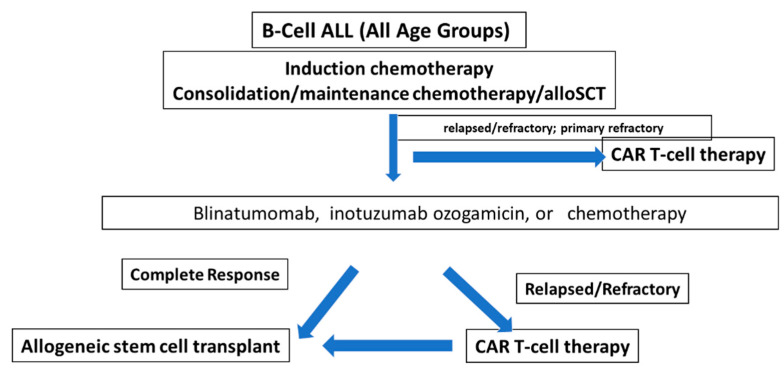
Fitting CAR T-Cell Therapy Into Current Treatment Paradigms in ALL.

In Figure 4, we postulate the introduction of CAR T-Cell therapy into current treatment paradigms in ALL.

## 3. Expert Opinion and Conclusions

CAR-T represents a clinical and technological revolution, showing that it is possible to genetically modify immune system cells to make them more effective against certain types of blood cancer. This approach has opened a new page in medicine because we have moved from the drug, an active ingredient ‘packaged’ and ready to be taken, to a highly personalized therapy. After administration, CAR-T remains active for some time, even for a long time, in more than half of the patients treated. The challenges of immunological therapies remain many. To overcome them, it is essential to make these therapies more effective, safe, and applicable to an ever-increasing number of diseases. Early enthusiasm should be tempered since several issues are still unsolved and represent challenges for the coming years.

A group of researchers evaluated CAR-T outcomes after failure of a previous allo-SCT, or without having had the allograft [53]. The results showed that the best responses to CAR-T were for patients who had not previously undergone allo-SCT. This conclusion must be carefully considered when planning CAR-T placement versus allograft. The limitation of all clinical research on CAR-T is that we only have data on a small and very select number of patients with restricted follow-up. In addition, the information we have comes from different CAR constructs, which could influence the outcomes. The main weakness is the lack of information on the immune status of patients who received CAR-T. The parameters of immunological functionality at the time of lymphocyte collection are the main factors influencing the efficacy of CAR-T. CAR-T-based therapies take lymphocytes from the patient, which are then modified in the laboratory and infused back into the circulation. While the treatment allows the toxicity of the T lymphocytes to be redirected, the starting lymphocyte capacity is maintained: the healthier the lymphocytes taken from the patient, the more efficient they can be after being engineered in the lab. allo-SCT before CAR-T therapy may, in this sense, decrease the effectiveness of the treatment because we are talking about patients who suffer severe immunosuppression following the administration of allogeneic stem cells. In some cases, lymphocytes are scarce, and in others they are not biologically adequate.

The immune system’s functionality is not the only factor influencing the efficiency of CAR-T. Before the re-infusion of engineered lymphocytes armed to fight tumor cells, the patient undergoes lymphodepleting chemotherapy treatment to reduce white blood cells and increase the likelihood of CAR-T cells taking root. The more intense the chemotherapy treatment, the greater is the probability of the cells taking root. Another factor influencing the efficiency of the treatment is the nature of the chimeric receptor used. Specific molecules with a costimulatory function introduced into the CAR construct can influence the possibility of expansion and survival of the engineered lymphocytes.

CAR-T and allo-SCT can cure hematological malignancies, but toxicity and failure represent two weaknesses. The adverse event profiles of CAR T-cell therapy and allo-SCT are different but with some overlaps. Both treatments can cause cytopenias, nausea, vomiting, fatigue, infections and the need for blood transfusions. However, some toxicities are very distinct and more reversible with CAR-T, and the treatment appears to have more manageable long-term toxicity, although follow-up is not yet adequate. Regarding relapse rates, CAR-T is less effective towards lymphoma, with a progression rate of around 60%. On the other side, TRM with allo-SCT is approximately 30%, and with CAR-T is less than 5%.

The clinical scenarios can be very different from each other: patients who receive allo-SCT, relapse, and then are cured with CAR T-cell therapies [54,55]; patients who do not benefit from either treatment; and patients who have received CAR T-cell therapies, relapse, then receive allo-SCT and are long-term survivors following allo-SCT. The therapeutic decision depends on the diagnosis. In lymphoma patients, it is reasonable to carry out CAR-T, assess the disease at 1–3 months, and if the patient is in CR, observe follow-up. If the patient is not in CR, alternative therapies, such as bispecific antibodies, should be evaluated as a bridge to allo-SCT. At this time, transplant-eligible patients who achieve a CR after CAR-T failure should be considered for allo-SCT. We also have to emphasize the need for long-term follow-up to confirm the duration of durable remission rates and the curative potential of allo-SCT after CAR-T cell failure.

The clinical landscape changes radically in ALL, where with the current state of treatment, achieving CR after CAR-T in R/R patients is not synonymous with cure [56]. In this context, there are two scenarios: CAR-T as a bridge or an alternative to allo-SCT [55]. The rationale for preferring one is summarized in Table 3, although there are few published data to select the best strategy. Current data on CAR-T are derived from phase I and II studies, and these trials focused on early responses and safety. There is a need for more information on critical aspects of CAR-T compared with data derived from allo-SCT studies. An issue is whether to consolidate a fit patient with an MRD-negative remission after CAR-T with allo-SCT. Some CAR T-cell products can have functional persistence in vivo that offers the potential for ongoing tumor surveillance, which could be lost if a patient receives allo-SCT [57].

CAR-T cells are exclusively granted to the use of autologous engineered lymphocytes. With the increasing use of CAR-T for relapse following allo-SCT and the imminence of allogeneic CAR-T, risks from T cell-based therapy, such as GVHD, have gained prominence and warrant explanation [58]. There is interest in developing ‘off-the-shelf’ allogeneic CAR-T for use outside of the context of a previous allo-SCT, as this would facilitate making CAR-T more widely available [59]. This is an active area of research where the risk of GVHD and graft rejection are major obstacles [60].

CAR-T represents a potential therapeutic option for patients in the post-allo-SCT setting [61]. In this setting, CAR-T may be manufactured from donor T cells (true allogeneic or donor CAR-T) or from the recipient’s T cells post-allo-SCT (pseudo-allogeneic or recipient CAR-T). However, GVHD remains a potential complication of allo-SCT, and allo-CAR T cells may increase the risk of developing de novo GVHD or exacerbating pre-existing GVHD [58].

In conclusion, it is desirable to know in the future the results of the long-term toxicity of CAR-T therapy, the efficacy after adequate follow-up, and the possible role and cost of subsequent treatment with allo-SCT (Table 4), particularly in patients in CR, and a negative MRD. Prospective studies tailored based on risk factors are needed to better define the optimal sequences of allo-SCT and cellular therapy and other approved novel therapies.

## Figures and Tables

**Table 1:** FDA and EMA-Approved CAR-T Cell Therapies.

Therapy	Construct	Dose	Lymphodepletion	Indications
**CD19-Targeting Therapies**				
Tisagenlecleucel	Anti–CD19-41BB-CD3z Uses lentiviral transduction	0.6 to 6.0 × 10^8^	Flu/Cy 25/250 × 3 days, or bendamustine × 2 days	▪ Patients aged up to 25 year with B-cell precursor ALL that is refractory or in second/later relapse ▪ Adults with R/R large B-cell lymphoma after ≥2 lines of systemic therapy, including DLBCL, NOS, DLBCL arising from follicular lymphoma, high-grade B-cell lymphoma ▪ Adults with R/R follicular lymphoma after ≥2 lines of systemic therapy
Axicabtagene ciloleucel	Anti–CD19-CD28-CD3z Uses retroviral transduction	2 × 10^6^/kg (max 2 × 10^8^)	Flu/Cy 30/500 × 3 days	▪ Adults with large B-cell lymphoma refractory to or relapsed within 12 months of first-line chemoimmunotherapy ▪ Adults with R/R large B-cell lymphoma after ≥2 lines of systemic therapy, including DLBCL NOS, DLBCL arising from follicular lymphoma, primary mediastinal large B-cell lymphoma, high-grade B-cell lymphoma ▪ Adults with R/R follicular lymphoma after ≥2 lines of systemic therapy
Brexucabtagene autoleucel	Anti–CD19-CD28-CD3z Uses retroviral transduction	2 × 106/kg (max 2 × 10^8^)	Flu/Cy 30/500 × 3 days	▪ Adults with R/R MCL ▪ Adults with R/R B-cell ALL
Lisocabtagene maraleucel	Anti–CD19-41BB-CD3z Uses lentiviral transduction	50 to 150 × 10^6^	Flu/Cy 30/300 × 3 days	▪ Adults with large B-cell lymphoma (including DLBCL NOS and DLBCL arising from indolent lymphoma) ▪ High-grade B-cell lymphoma, primary mediastinal large B-cell lymphoma and follicular lymphoma grade 3B that is: - refractory to or relapsed within 12 mo of first-line chemoimmunotherapy - R/R after first-line chemoimmunotherapy and not eligible for auto-SCT due to comorbidities or age - R/R after ≥2 lines of systemic therapy
**BCMA-Targeted Therapies**				
Idecabtagene vicleucel	Anti–BCMA-41BB-CD3z Uses lentiviral transduction	150 to 450 × 10^6^	Flu/Cy 30/300 × 3 days	▪ Adults with R/R MM after ≥4 prior lines of therapy, including an immunomodulatory agent, a proteasome inhibitor, and an anti-CD38 monoclonal antibody
Ciltacabtagene autoleucel	Anti–BCMA-41BB-CD3z Uses lentiviral transduction	0.5–1 × 10^6^	Flu/Cy 30/300 × 3 days	

Legend: diffuse large B-cell lymphoma = DLBCL; not otherwise specified = NOS; relapsed or refractory = R/R; B-cell maturation antigen = BCMA; fludarabine/cyclophosphamide = Flu/Cy; acute lymphoblastic leukemia = ALL; mantle cell lymphoma = MCL; multiple myeloma = MM; autologous stem cell transplantation = auto-SCT.

**Table 2:** Pivotal Anti-CD19 CAR T-Cell Therapy Trials: DLBCL.

	JULIET	ZUMA-1	TRANSCEND NHL 001
CAR T-cell agent	Tisa-cel	Axi-cel	Liso-cel
Patient population	Adults with R/R DLBCL post/ineligible for Auto-SCT	Adults with R/R DLBCL	Adults with R/R DLBCL
Patients apheresed/treated, n	165/111	111/101	344/269
ORR, % CR, %	52% 40%	82% 54%	73% 53%
Survival	12-mo PFS 65% 12-mo OS 49%	PFS 5.8 mo 18-mo OS 52%	12-mo RFS 44% 12-mo OS 58%

Legend: diffuse large B-cell lymphoma = DLBC; relapsed/refractory = R/R.

**Table 3:** The rationale to select the best therapy in all patients.

CAR-T Approach	Rational	Advantages	Disadvantage
Bridge to Transplant	Used as a bridge to allo-SCT to induce deep remissions in R/R patients.	Highly effective immunological therapies, recognizing the role of allo-SCT as a standard of care in the treatment for high-risk R/R ALL Determine the patient’s clinical remission, which is a key surrogate for optimizing transplant response	allo-SCT toxicity, The high price of two therapies More readily available, practical, and less expensive bridging agent alternatives to CAR-T (i.e., blinatumomab, Inotuzumab) Unclear applicability in patients who have been transplanted before and are not eligible for a second allo-SCT
Alternative to allo-SCT	Based on CAR T cells as a stand-alone treatment to replace allo-SCT.	Applicability to refractory patients and those with active or progressing disease No donor requirement Logistically easier More feasible in elderly patients Avoidance of toxic short and long-term allo-SCT complications	Long-lasting B Cell Aplasia as an on-target effect of B-cell-targeting CAR-T. The long-term effects of CAR-T- on the immune system need further observation. CAR-T targeting single antigens risks provoking target-negative subclones that the broader graft-vs.-leukemia effect of a consolidative allo-SCT post-CAR-T could potentially eliminate. Multi-antigen targeting approaches may overcome tumor escape in a CAR-T stand-alone strategy.

**Table 4:** Points of future discussion between CAR-T and allo-SCT.

Safety	Long-Term Trials to Detect Toxicities That Might Appear with More Extended Observation Times Post Infusion
Efficacy	Long-term efficacy of CAR-T, focusing on therapies given in addition to the initial CAR T-cell infusion (e.g., checkpoint inhibitors, targeted molecular therapies such as tyrosine kinase inhibitors, and consolidative allo-SCT while patients are still in remission)
Cost	Strategies based on sequential therapies (e.g., CAR-T followed by allo-SCT; allo-SCT followed by CAR-T; CAR-T followed by CAR-T; CAR-T followed by allo-SCT followed by CAR-T, etc.)

## Data Availability

Not applicable.
